# The Over-Irradiation Metabolite Derivative, 24-Hydroxylumister-ol_3_, Reduces UV-Induced Damage in Skin

**DOI:** 10.3390/metabo13070775

**Published:** 2023-06-21

**Authors:** Warusavithana Gunawardena Manori De Silva, Bianca Yuko McCarthy, Jeremy Han, Chen Yang, Andrew J. A. Holland, Harvey Stern, Katie Marie Dixon, Edith Kai Yan Tang, Robert Charles Tuckey, Mark Stephen Rybchyn, Rebecca Sara Mason

**Affiliations:** 1 School of Medical Sciences and Bosch Institute, The University of Sydney, Sydney, NSW 2006, Australia; wdes3780@uni.sydney.edu.au (W.G.M.D.S.); bianca.mccarthy@lubrizol.com (B.Y.M.); j.han@garvan.org.au (J.H.); ayang@cmri.org.au (C.Y.); katie.dixon@sydney.edu.au (K.M.D.);; 2 Douglas Cohen Department of Paediatric Surgery, The Children’s Hospital at Westmead Clinical School, The Faculty of Medicine and Health, The University of Sydney, Sydney, NSW 2006, Australia; andrew.holland@health.nsw.gov.au; 3 Department of Plastic and Constructive Surgery, The Royal Prince Alfred Hospital, Sydney, NSW 2050, Australia; hstern@bigpond.net.au; 4 Strathfield Private Hospital, Sydney, NSW 2042, Australia; 5 School of Molecular Sciences, The University of Western Australia, Perth, WA 6009, Australia; edith.tang@uwa.edu.au (E.K.Y.T.); robert.tuckey@uwa.edu.au (R.C.T.); 6 School of Life and Environmental Sciences, Charles Perkins Centre, University of Sydney, Sydney, NSW 2006, Australia

**Keywords:** 1,25(OH)_2_D_3_,1,25-dihydroxyvitamin D_3_, 24(OH)L_3_, 24-hydroxy-lumisterol_3_, 8OHdG, 8-hydroxy-2′-deoxyguanosine, ATP, adenosine tri-phosphate, CPD, cyclobutane pyrimidine dimer, CYP11A, cytochrome P450 side-chain cleavage enzyme 11A, ROS, reactive oxygen species, siRNA, small interfering RNA, UVR, ultraviolet radiation, UDS, unscheduled DNA synthesis, VDR, vitamin D receptor, XPA, xeroderma pigmentosum complementation group A, XPC, xeroderma pigmentosum complementation group C

## Abstract

The hormonal form of vitamin D_3_, 1,25(OH)_2_D_3_, reduces UV-induced DNA damage. UV exposure initiates pre-vitamin D_3_ production in the skin, and continued UV exposure photoisomerizes pre-vitamin D_3_ to produce “over-irradiation products” such as lumisterol_3_ (L_3_). Cytochrome P450 side-chain cleavage enzyme (CYP11A1) in skin catalyzes the conversion of L_3_ to produce three main derivatives: 24-hydroxy-L_3_ [24(OH)L_3_], 22-hydroxy-L_3_ [22(OH)L_3_], and 20,22-dihydroxy-L_3_ [20,22(OH)L_3_]. The current study investigated the photoprotective properties of the major over-irradiation metabolite, 24(OH)L_3_, in human primary keratinocytes and human skin explants. The results indicated that treatment immediately after UV with either 24(OH)L_3_ or 1,25(OH)_2_D_3_ reduced UV-induced cyclobutane pyrimidine dimers and oxidative DNA damage, with similar concentration response curves in keratinocytes, although in skin explants, 1,25(OH)_2_D_3_ was more potent. The reductions in DNA damage by both compounds were, at least in part, the result of increased DNA repair through increased energy availability via increased glycolysis, as well as increased DNA damage recognition proteins in the nucleotide excision repair pathway. Reductions in UV-induced DNA photolesions by either compound occurred in the presence of lower reactive oxygen species. The results indicated that under in vitro and ex vivo conditions, 24(OH)L_3_ provided photoprotection against UV damage similar to that of 1,25(OH)_2_D_3_.

## 1. Introduction

While sunlight clearly has many positive benefits, the detrimental effects of sun exposure on skin were first proposed by Paul Gerson Unna in 1894 [1]. Studies since then have shown that UVB radiation in sunlight induces harmful effects such as sunburn, DNA damage [2,3], cell-mediated immunosuppression [4], mutations, and photocarcinogenesis [5]. UVA also contributes to DNA damage and photo-aging [6,7]. The harmful effects of UVA and UVB both contribute to photocarcinogenesis.

When sequential pyrimidine bases of the same DNA strand absorb UV photons, this direct DNA damage results in dimeric photolesions [3,8]. *Cis-syn* cyclobutane pyrimidine dimers (CPDs), mostly formed between 5–6 bonds of adjacent thymine and cytosine bases, are the most common lesions found. Of these, thymine dimers (T–T) are the majority and are formed in numbers directly proportional to the total of all types of CPDs [9]. Thymine–cytosine dimers and cytosine–cytosine dimers have been shown to be highly mutagenic [2,10].

Indirect DNA damage such as oxidative damage to DNA purine bases contributes to mutagenesis, aging, pathological conditions, and photocarcinogenesis [11,12,13]. Indirect DNA damage is mainly caused by free radicals, reactive oxygen species (ROS) [14,15], and reactive nitrogen species (RNS) [16]. UV exposure increases ROS production and depletes antioxidants, resulting in an increased ROS to antioxidant ratio, considered to be the main contributor to the formation of oxidative photolesions [17,18,19,20]. Types of ROS include singlet oxygen (^1^O_2_) [15], hydroxyl radical, hydrogen peroxide, and the superoxide anion [21,22]. These free radicals mainly target guanine in the DNA, and this oxidation reaction produces the main photolesion, 8-oxo-7, 8-dihydro-2′-deoxyguanosine (8-OHdG) [23,24,25,26,27]. ROS contributes to gene mutations [28], DNA strand breakage [29], aging [30], mitochondrial damage [31], structural damage (organelle membranes), and functional protein damage (including DNA repair proteins), all of which may promote carcinogenesis [32,33,34,35].

CPDs are repaired by the highly conserved nucleotide excision repair (NER) pathway [36,37,38]. Two subpathways of NER consist of transcription coupled nucleotide excision repair (TC-NER) and global genomic nucleotide excision repair (GG-NER). The major difference between these pathways lies in the initial damage recognition process. One important damage recognition protein involved in GG-NER is xeroderma pigmentosum complementation group C (XPC) protein [39,40,41]. XPC acts as the main damage sensor that triggers GG-NER [42,43] and is a structure-specific damage factor [44,45]. The xeroderma pigmentosum complementation group A (XPA) protein has a higher affinity for damaged DNA than undamaged DNA [46] and is vital for progression in both TC-NER and GG-NER [47].

**Figure 1:**
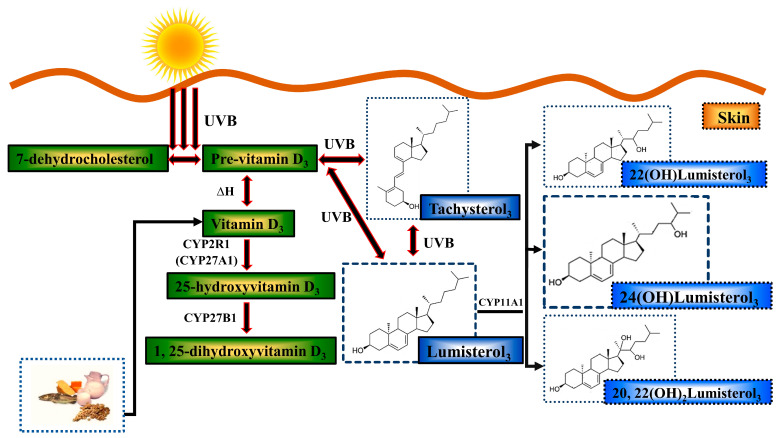
Biosynthesis and metabolism of vitamin D and over-irradiation products after UV radiation. When 7-dehydrocholesterol in skin absorbs UVB, it is converted to pre-vitamin D_3_. Pre-vitamin D_3_ thermo-isomerizes into vitamin D_3_ at body temperature. Vitamin D is converted in the liver and other tissues, including skin, to 25-hydroxyvitamin D_3_ by CYP2R1/CYP27A1 enzymes and then to the biologically active vitamin D hormone, 1,25-dihydroxyvitamin D_3_ (1,25(OH)_2_D_3_), in the kidney and other tissues including skin by CYP27B1. Exogenous vitamin D can be acquired through food, such as fish or supplements. Continuous UVB exposure photo-isomerizes pre-vitamin D_3_ to produce what used to be considered relatively biologically inactive “over-irradiation products” such as lumisterol_3_ (L3), tachysterol_3_, and other compounds. CYP11A1 is a cytochrome P450 enzyme found in skin which catalyzes L3 to 22(OH)lumisterol_3_, 24(OH)lumisterol_3_, and 20,22(OH)_2_lumisterol_3_ as the major products. Green color shows classic vitamin D compounds. Blue color shows over-irradiation products and metabolites.

One of the beneficial effects of UVB exposure is the biosynthesis from 7-dehydrocholesterol of pre-vitamin D_3_, which at body temperature isomerizes to vitamin D_3_ [48]. Skin exposed to UVB radiation converts vitamin D_3_ to its hormonal form, 1,25(OH)_2_D_3_ (Figure 1) [49,50,51]. Several studies have shown that vitamin D metabolites reduce UV-induced DNA damage in skin cell cultures, mice, and human skin [52,53,54,55,56,57]. This reduction in DNA damage leads to reductions in skin tumors in mouse models [53,57].

Vitamin D_3_ is not the only compound produced in skin from 7-dehydrocholesterol by UVB exposure. Also produced are the so-called “over-irradiation” products [58,59,60] (Figure 1). Continued exposure to UV photoisomerizes pre-vitamin D_3_ to produce biologically “inactive over-irradiation products”, lumisterol_3_ and tachysterol_3_ [61,62,63,64]. Cytochrome P450 side-chain cleavage enzyme/CYP11A1 is a mitochondrial enzyme [65], which upregulates after UVB exposure [66,67,68]. This enzyme catalyzes the conversion of vitamin D_3_ itself to several isomers that have some photoprotective properties [69,70,71,72,73] and converts lumisterol_3_ into 24-hydroxylumisterol_3_ [24(OH)L_3_], 22-hydroxylumisterol_3_ [22(OH)L_3_], and 20,22-dihydroxylumisterol_3_ [20,22(OH)_2_L_3_] [68,72]. Lumisterol_3_ and its hydroxyderivatives are metabolically stable in that they are not metabolized by 7-dehydrocholesterol reductase, unlike their 7-dehydrocholesterol stereoisomers [74]. The current study investigated the in vitro and ex vivo photoprotective properties on DNA damage of the over-irradiation metabolite, 24(OH)L_3_.

## 2. Materials and Methods

### 2.1. Cell Cultures and Skin Explants

Skin samples for keratinocyte culture or skin explant culture were harvested after obtaining written informed consent from subjects or their parents/guardians, with approval from the University of Sydney Human Ethics Committee (Reference number 2015/063).

- Keratinocytes: Keratinocytes were grown out from skin fragments [75] and cultured as previously described with minor modifications [76], in keratinocyte growth medium (KGM), which contained minimum essential medium Eagle (M0518, Sigma-Aldrich, St. Louis, MO, USA), 0.02 M sodium bicarbonate (Sigma-Aldrich, St. Louis, MO, USA), 1 mM sodium pyruvate, and 25 mM HEPES (ThermoFisher Scientific, Waltham, MA, USA) in MilliQ water at pH 7.2 (Millipore SAS, Molsheim, France). Human primary keratinocytes were cultured in KGM containing 5% (*v*/*v*) fetal calf serum (FCS) and the following supplements: 5 μg/mL transferrin, 0.4 ug/mL hydrocortisone, 1 × 10^−10^ M cholera toxin, 10 ng/mL Epidermal Growth Factor (EGF), 5 μg/mL insulin (all from Sigma-Aldrich, St. Louis, MO, USA), and 2 × 10^−11^ M 3,3,5-triiodo-L-thyronine sodium salt [77]. Keratinocytes from passages 2–5 from at least two independent donors were used in all experiments. The keratinocyte culture media was changed to media without EGF and cholera toxin for 24 h before experiments to allow cells to become quiescent [78].
- Skin explants: Human skin explants were collected from consenting patients undergoing elective surgery at private hospitals in Sydney, Australia, and processed as previously described [56]. In brief, ice-cold sterile phosphate buffered saline (PBS) was used to transport the skin back to the laboratory. The skin was processed under aseptic conditions within 4 h of surgery. The skin was briefly washed with 4% chlorhexidine gluconate solution (Sigma-Aldrich, St. Louis, MO, USA) and then thoroughly washed with ice-cold PBS. Subcutaneous fat and debris were trimmed off to leave the epidermis and dermis only, for the study. The skin samples were dissected into 4 mm pieces with a punch biopsy tool with five biopsies prepared for each treatment. The skin samples were then prepared for UV irradiation as described below.

### 2.2. Solar-Simulated UV Irradiation

UV irradiation was provided by an Oriel Sol1A^TM^ 94042A 450 W solar simulator (Newport Corporation, Irvine, CA, USA) with an atmospheric attenuation filter to eliminate UVC (<290 nm). It was calibrated using the OL756 spectroradiometer (Gooch & Housego, Melbourne, FL, USA), and an IL 1700 broadband radiometer (International Light Technologies, Peabody, MA, USA) was used immediately prior to experiments to determine the UVB output, from which a calculated dose of solar-simulated UV (ssUV) was determined. This dose of UV did not increase caspase activity or decrease the total DNA and equated to approximately 4 min of sunlight at noon in October in Sydney, Australia [79]. The spectral output of this lamp as used for these experiments has been published [Figure S6 in [79]].

- Keratinocytes: Immediately prior to UV irradiation, the medium was replaced with irradiation buffer, Martinez solution containing 10 mM D-glucose (Sigma-Aldrich, St. Louis, MO, USA) without phenol red. For all keratinocyte experiments, the ssUV irradiation energy level was 400 mJ/cm^2^ UVB and 3600 mJ/cm^2^ UVA (4000 mJ/cm^2^) [53,79].
- Skin explants: Skin biopsies were placed in ice-cold sterile colorless Martinez buffer with the epidermis facing up in a volume that was just enough to surround the tissue without submersion, for UV radiation [56]. A single dose of ssUV at 20 J/cm^2^ was delivered to the skin explant samples. Sham/non-irradiated keratinocytes or skin were subjected to similar procedures but not irradiated.

### 2.3. 1,25( OH)_2_D_3_ and 24(OH)L_3_ Treatments

The 1,25(OH)_2_D_3_ was from Sapphire Bioscience Pty Ltd., (Sydney, Australia). The 24(OH)L_3_ was enzymatically synthesized using recombinant CYP11A1 and purified using procedures as previously described [72,80]. These compounds were solubilized in spectroscopic grade ethanol (Merck, Darmstadt, Germany) and added to cultures in 0.1% (*v*/*v*) ethanol. The ethanol vehicle 0.1% (*v*/*v*) was used as a control. The concentration of these compounds was determined with a Nanodrop ND-1000 Spectrophotometer (ThermoFisher Scientific, Waltham, MA, USA) immediately prior to use.

- Keratinocytes: Immediately after UV irradiation (or sham irradiation), the irradiation buffer was replaced with supplement-free keratinocyte growth medium (KGM) containing vehicle, 0.1% (*v*/*v*) ethanol, or treatments at the concentrations as indicated.
- Skin explants: Immediately after UV irradiation (or sham irradiation), the skin samples were treated with vehicle, 0.1% (*v*/*v*) ethanol, or treatments in RPMI-1640 media (Sigma-Aldrich, St. Louis, MO, USA) supplemented with 10% FCS, penicillin, and streptomycin at 37 °C.

### 2.4. Immunohistochemistry

The current study used an antibody detection method for thymine dimers as an index of total CPDs and antibody detection of 8-OHdG followed by image analysis. These methods produced similar results to those that detected CPDs or 8-OHdG by a specific endonuclease followed by a Comet assay [54,76]. CPDs consist of thymine–thymine, thymine–cytosine, cytosine–thymine, or cytosine–cytosine photoproducts [81]. Using a direct assay of a combination of liquid chromatography and tandem mass spectrometry to determine the different fractions of dimer photoproducts after UV, thymine dimers were found to be the most abundant, eightfold higher than other photolesions and correlated with the number of other dimers [9,82]. Thus, thymine dimers are routinely used as a measure of the total CPDs.

- Keratinocytes:

CPDs and 8-OHdG: At the end of the incubation period after UV exposure (3 h or the period mentioned for the experiment), the keratinocytes were rinsed with PBS after incubation, and the keratinocytes were fixed with ice-cold 100% methanol (Sigma-Aldrich, St. Louis, MO, USA) at −20 °C for 10 min. Cells were extensively washed with MilliQ water and air dried overnight prior to immunohistochemistry, which was carried out as previously described [55], with minor modifications. Endogenous peroxidase activity was blocked by 1% (*v*/*v*) H_2_O_2_ (ThermoFisher Scientific, Waltham, MA, USA) in PBS for 5 min followed by two MilliQ water washes. Antigen retrieval was carried out in several steps. Nuclear DNA was denatured with 70 mM NaOH (Sigma-Aldrich, St. Louis, MO, USA) in 70% ethanol for 2 min. Proteolytic digestion was achieved with 1 μg/mL Proteinase K (Sigma-Aldrich, St. Louis, MO, USA) in 0.1 mM CaCl_2_ for 5 min for CPD detection and 10 min for 8-OHdG detection followed by two MilliQ water washes. Non-specific antibody binding was inhibited by treating cells with 50% (*v*/*v*) horse serum (Sigma-Aldrich, St. Louis, MO, USA) in PBS (blocker) for 1 h. The cells were incubated with primary antibody for 60 min at room temperature, either mouse monoclonal anti-thymine dimer at 5 µg/mL (Sigma-Aldrich, St. Louis, MO, USA) for CPDs or primary mouse monoclonal IgG_2b_ anti-8-hydroxy-guanosine antibody (Santa Cruz Biotechnologies, Dallas, TX, USA) for 8-OHdG at 2.5 µg/mL or an equivalent concentration of isotype control, followed by three TBS-T washes. The cells were incubated with biotinylated goat anti-mouse IgG secondary antibody (ThermoFisher Scientific, Waltham, MA, USA) diluted to 1:500 in Tris-buffered saline with 0.1% Tween 20 (TBS-T) for 15 min followed by three TBS-T washes. Horseradish peroxidase (HRP)-Streptavidin Conjugate (Invitrogen, Waltham, MA, USA) diluted 1:150 in TBS-T was added for 15 min followed by three TBS-T washes. The HRP substrate Diaminobenzidine (DAB) (Enhanced Liquid substrate System for Immunohistochemistry, Sigma-Aldrich, St. Louis, MO, USA) was added for 5 min followed by two MilliQ water washes. Isotype antibodies were run as controls for all experiments, but as previously published, the isotype controls resulted in minimal staining [83]. The coverslips were mounted on glass slides with entellen rapid mounting medium (Merck, Darmstadt, Germany) for image analysis [55].

- Skin explants:

After 3 h incubation, skin samples were fixed in 10% (*v*/*v*) neutral buffered formalin overnight, then paraffin-embedded and sectioned. The paraffin-embedded sections were first deparaffinized in xylene (ThermoFisher Scientific, Waltham, MA, USA), then hydrated by passing the slides through graded alcohol. Antigen retrieval was carried out by treating sections with citrate buffer, pH 6, at 95 °C for 30 min as previously described [56].

CPDs or 8-OHdG: Only for 8-OHdG staining, sections were incubated with RNase A (AMRESCO, Cleveland, OH, USA) at 37 °C for 30 min to remove 8-OHdG that would otherwise be detected in cellular RNA [84], followed by two TBS-T washes. For both lesions, sections were treated with 2 N HCl (Sigma-Aldrich, St. Louis, MO, USA) in 70% ethanol for 15 min at room temperature to denature DNA and further expose damaged sites. Following aspiration, the residual acid solution was neutralized with 50 mM Tris-HCl (pH 7.5) for 15 min at room temperature, followed by two further TBS-T washes. Sections were then blocked with 10% (*v*/*v*) horse serum in PBS, pH 7.2, for 60 min at room temperature, then incubated with primary antibody for 60 min at room temperature, either mouse monoclonal anti-thymine dimer at 10 µg/mL for CPDs or primary mouse monoclonal IgG_2b_ anti-8-hydroxy-guanosine antibody at 2.5 µg/mL for 8-OHdG or an equivalent concentration of isotype control, followed by three TBS-T washes.

*XPC* or *XPA*: Following the antigen retrieval, the XPC and XPA sections were blocked with avidin and biotin from the Dako Cytomation Blocking System (Dako, Glostrup, Denmark) for 10 min at room temperature with a brief rinse in between treatments, followed by blocking with 10% (*v*/*v*) horse serum in PBS for 60 min at room temperature. The sections were incubated with primary antibodies overnight at 4 °C, either with mouse monoclonal IgG_2a_ XPC at 2.5 µg/mL, mouse monoclonal IgG_1_ XPA antibody at 4.0 µg/mL (both from Santa Cruz Biotechnology, Dallas, TX, USA), or an equivalent concentration of isotype control, followed by three TBS-T washes.

Detection of antibody binding: This was carried out for all sections with the Dako LSAB Plus REAL Detection System Kit with AEC chromogen staining, using the methods provided by the manufacturer (Dako, Glostrup, Denmark). The slides were mounted with DPX (Sigma-Aldrich, St. Louis, MO, USA) and coverslipped.

### 2.5. Image Acquisition

- Keratinocytes: Bright-field images were acquired on the Olympus stereo investigator scope (MBF Bioscience, Williston, VT, USA) or on the Zeiss AxioScan-Z1 slide scanner (Zeiss Microscopy, Oberkochen, Germany) at the Bosch Advanced Microscopy Facility (University of Sydney). All images were taken at 20× magnification, and the immunohistochemical images were analyzed using ImageJ software. Using this program, images from an individual experiment were thresholded to the same value. The regions of interest (ROI) were randomly selected. The means and standard errors of the mean (SEMs) from three to five ROI per coverslip of each treatment were calculated and graphed. Staining and image analysis for CPDs and 8-OHdG produced similar results to those obtained using endonuclease detection of the lesion, followed by Comet assay [54,76].
- Skin explants: Bright-field images were captured using the Zeiss Axio Scan (Zeiss Microscopy, Oberkochen, Germany). For each section, images of the whole skin section were taken at 20× magnification. All images were analyzed using MetaMorph imaging software (Molecular Devices Corporation, San Jose, CA, USA), whereby the epidermal area was isolated and thresholded specific for positive nuclei in this region. This software then automatically calculated the positive nuclei as a percentage of the total epidermal area.

### 2.6. siRNA Transfection

Plated keratinocytes were transfected with 50 nM siRNA directed at XPC (siXPC), XPA (siXPA), or a control non-directed sequence (siCTRL) (all from Santa Cruz Biotechnologies, Dallas, TX, USA), following combination with a lipid-based transfection reagent (Santa Cruz Biotechnologies, USA) in Opti-MEM™ serum-free media (ThermoFisher Scientific, MA, USA) according to the manufacturer’s instructions and as previously described [85]. The cells were incubated overnight, and on the following day, an equal volume of 10% (*v*/*v*) FCS in KGM without EGF and cholera toxin was added to deliver a final concentration of 5% (*v*/*v*) KGM without EGF and cholera toxin. The cells were further incubated overnight under standard culture conditions prior to irradiation and treatment.

### 2.7. Western Blot

Keratinocytes were plated directly in six-well plates in KGM with supplements. Following the indicated treatments, the cells were lysed and subjected to Western blot as previously described [79] with minor modifications. The transfer of protein to a nitrocellulose membrane (AmershamTM Protran 0.45 lm NC; GE Healthcare) was achieved at 25 V overnight at 4 °C. The membrane was then blocked with 5% (*w*/*v*) BSA in TBS-T at pH 7.2 for 1 h before being incubated with mouse monoclonal IgG_2a_ XPC (Santa Cruz Biotechnologies, CA, USA) at 1 μg/mL in 5% (*w*/*v*) BSA in TBS-T, overnight at 4 °C. The following day, the membrane was washed with TBS-T three times and incubated with secondary goat anti-mouse IgG HRP-linked antibody (Cell Signaling Technology, Danvers, MA, USA) in 5% (*w*/*v*) BSA in TBS-T for 1 h at room temperature. After incubation, the membrane was washed three times with TBS-T before adding chemiluminescence substrate (Millipore SAS, Molsheim, France) for band detection. The bands were imaged with the ChemiDocTM imaging system (Bio-Rad Laboratories, Inc., Hercules, CA, USA) at the Bosch Research Institute (University of Sydney), and densitometry was carried out using ImageJ software (version 1.50).

### 2.8. Unscheduled DNA Synthesis

This was carried out as previously described [79]. In brief, EDU (5-ethynyl-2′-deoxyuridine) incorporation into nuclear DNA was detected using the iClick^TM^ EdU Andy Fluro 488 Imaging Kit (GeneCopoeia Inc, Rockville, MD, USA). The incorporation of this thymidine analog in a punctate pattern, in keratinocytes cultured without growth factors EGF and cholera toxin, measured unscheduled DNA synthesis rather than DNA replication [86]. Densitometry was performed using ImageJ software. The stained cells were manually counted from the images taken with a Zeiss LSM 510 Meta confocal microscope (Zeiss Microscopy, Oberkochen, Germany). The fluorescence intensity was measured in cells that were above the detection threshold and considered “UDS positive”, and fluorescence images were acquired by confocal microscopy and analyzed using ImageJ software [79].

### 2.9. Seahorse Energetics

An XF96 Extracellular Flux Analyzer (Seahorse Bioscience, Agilent, CA, USA) at Bosch Research Institute, University of Sydney, was used immediately following irradiation of cells to measure ECAR, as previously described [79].

### 2.10. ATP Measurement

ATP levels were determined using the CellTiter-Glo^®^ 2.0 Assay (Promega Corporation, Madison, WI, USA) as previously described [79].

### 2.11. ROS Measurement

ROS levels were measured using the ROS-Glo™ H2O2 assay (Promega Corporation, WI, USA) as previously described [79].

### 2.12. Statistical Analysis

- In vitro: Three independent experiments with triplicates per each treatment group were performed for each study with similar results, using keratinocytes from different donors. Unless otherwise indicated, the analyses were carried out by ANOVA with Tukey multiple comparisons post-test (GraphPad Prism statistical program) (CA, USA).
- Ex vivo: Three independent experiments were performed for each study with skin from different donors with similar results. Comparisons between treatments were made by one-way ANOVA followed by Sidak’s multiple comparisons test, using the GraphPad Prism statistical program (CA, USA), unless otherwise stated. The data in the graphs represent the mean + SEM, unless otherwise stated.

## 3. Results

### 3.1. 24(OH)L_3_ Reduced UV-Induced CPDs and 8-OHdG in a Concentration-Dependent Manner, Similar to 1,25(OH)_2_D_3_ in Human Primary Keratinocytes

**Figure 2:**
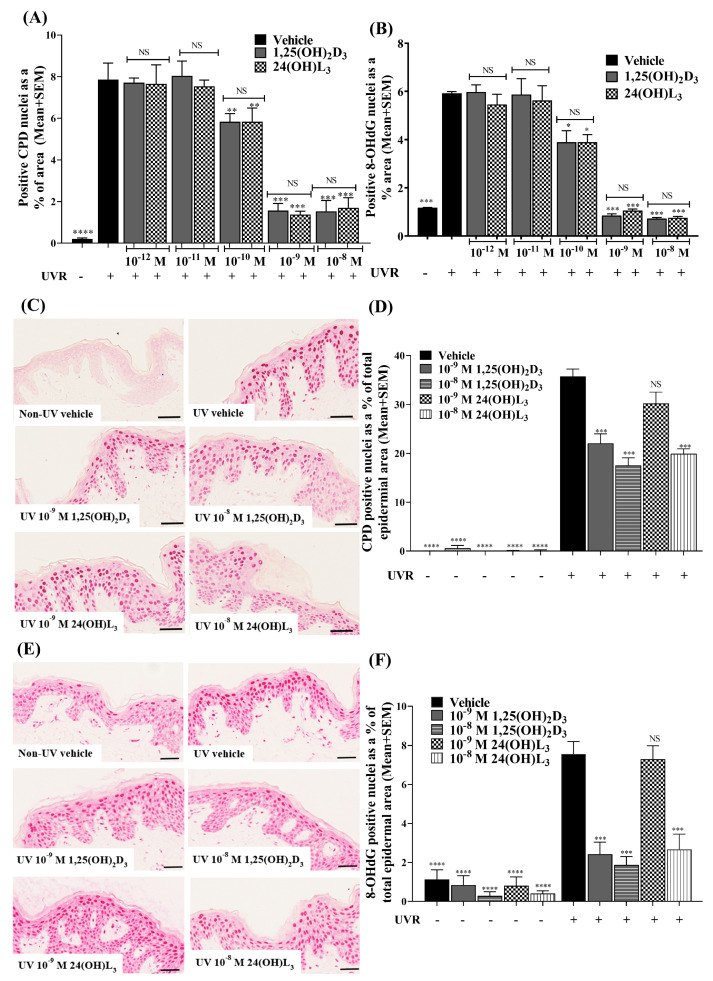
Concentration-dependent reduction of UV-induced CPDs or 8-OHdG by 1,25(OH)_2_D_3_ or 24(OH)L_3_ in human primary keratinocytes and human skin explants. Human keratinocytes or skin explants were exposed to solar simulated UV and then treated immediately after with vehicle, 0.1% (*v*/*v*) ethanol, or the positive control, 1,25(OH)_2_D_3_, or 24(OH)L_3_ at five different concentrations (keratinocytes) or 1 × 10^−9^ M or 1 × 10^−8^ M (skin explants) for 3 h after irradiation, followed by immunohistochemical staining for CPDs (**A**,**C**,**D**) or 8-OHdG (**B**,**E**,**F**) photolesions. Densitometry of keratinocytes is presented as (**A**) CPD-positive nuclei as a % area and (**B**) 8-OHdG-positive nuclei as a % area. Results for keratinocytes are presented as mean + SEM from a single experiment performed in triplicate, representative of three separate experiments with similar results. Representative photomicrographs of immunohistochemical staining of UV-induced (**C**) CPDs and (**D**) 8-OHdG (scale bar = 50 µm). Metamorph analysis of IHC images of skin explants presented as (**E**) CPD- and (**F**) 8-OHdG-positive nuclei as percentage of total epidermal area. Results are from a single experiment performed with five explants per group. Presented as mean + SEM; * *p* < 0.05, ** *p* < 0.01, *** *p* < 0.001, **** *p* < 0.0001, when compared with UV vehicle; NS—not significant between datasets.

UV-induced CPDs and 8-OHdG were increased in UV-irradiated vehicle-treated cells compared with non-UV-irradiated cells (Figure 2A,B; *p* < 0.0001, *p* < 0.001). In UV-irradiated keratinocytes, treatment with either 24(OH)L_3_ or 1,25(OH)_2_D_3_ significantly reduced UV-induced CPDs (*p* < 0.01) (Figure 2A) and 8-OHdG (*p* < 0.05) (Figure 2B) at 1 × 10^−10^ M and higher concentrations (*p* < 0.001).

### 3.2. 24(OH)L_3_ Reduced UV-Induced CPDs and 8-OHdG at Higher Concentrations Than 1,25(OH)_2_D_3_ in Human Skin Explants

Very low levels of CPDs (Figure 2C,D) and 8-OHdG (Figure 2E,F) were observed in non-UV-exposed skin explants compared with UV-irradiated vehicle-treated human skin explants (*p* < 0.0001). For the skin explants treated immediately after UV irradiation with either 1 × 10^−9^ M or 1 × 10^−8^ M 1,25(OH)_2_D_3_, there were significant reductions in UV-induced CPDs (Figure 2C,D; *p* < 0.001) and 8-OHdG (Figure 2E,F; *p* < 0.001). Treatment with 1 × 10^−8^ M 24(OH)L_3_ also significantly reduced both CPDs and 8-OHdG (*p* < 0.001 for both), but no significant reductions in either type of DNA damage were observed after treatment with 1 × 10^−9^ M 24(OH)L_3_ (Figure 2C–F).

### 3.3. 24(OH)L_3_ Increased Unscheduled DNA Synthesis Similar to 1,25(OH)_2_D_3_ in UV-Irradiated Human Primary Keratinocytes

**Figure 3:**
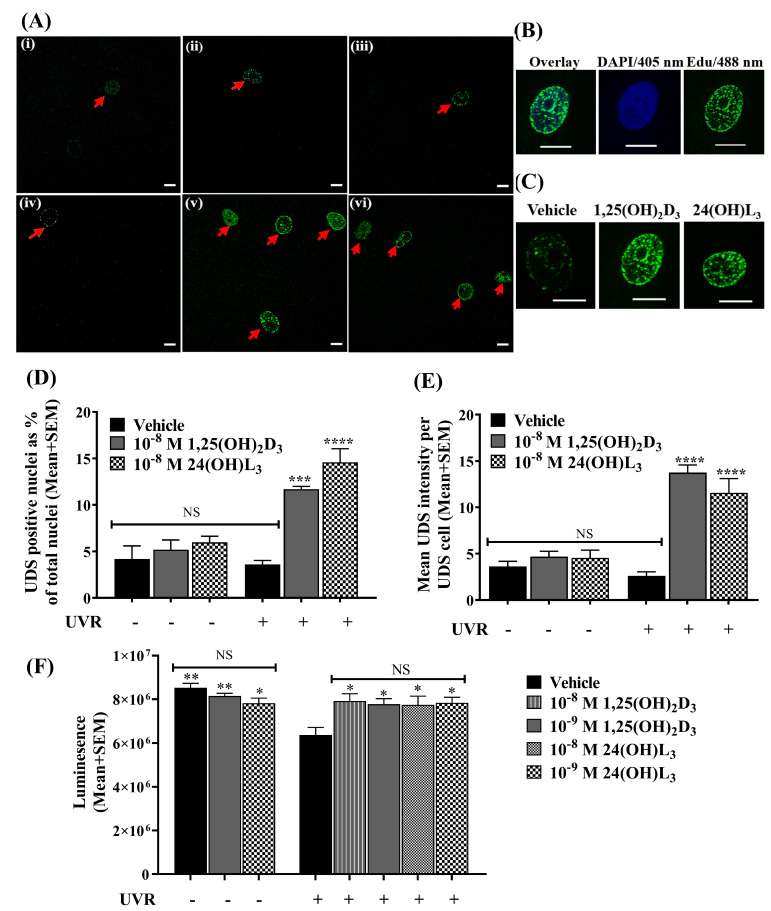
Unscheduled DNA synthesis (UDS) and ATP levels increased in human primary keratinocytes after UV exposure and treatment with 1,25(OH)_2_D_3_ or 24(OH)L_3_ following UV irradiation. (**A**) Example confocal images for incidence of UDS-positive nuclei: (**i**) non-UV vehicle, 0.1% (*v*/*v*) ethanol; (**ii**) non-UV 1,25(OH)_2_D_3_ (1 × 10^−8^ M); (**iii**) non-UV 24(OH)L_3_ (1 × 10^−8^ M); (**iv**) UV vehicle, 0.1% (*v*/*v*) ethanol; (**v**) UV 1,25(OH)_2_D_3_ (1 × 10^−8^ M); and (**vi**) UV 24(OH)L_3_ (1 × 10^−8^ M) (scale = 10 µm). (**B**) Example confocal images of UDS-positive nuclei showing EDU incorporation with DAPI counterstain (EDU incorporation shown by punctate nuclei) (scale = 10 µm). (**C**) Example confocal images of EDU intensity in UV-irradiated cells treated with vehicle, 1,25(OH)_2_D_3_, or 24(OH)L_3_. (**D**) UDS-positive nuclei as a percentage of total nuclei, for randomly counted 1000 nuclei per treatment group, 1.5 h after UV irradiation. (**E**) Average EDU intensity per UDS-positive cell nuclei (15 nuclei for each treatment). (**F**) ATP levels measured by CellTiterGlo 2.0 assay at 1.5 h after UV exposure and treatments. Results were from a single experiment performed in triplicate, representative of two separate experiments with similar results, presented as mean + SEM; * *p* < 0.05, ** *p* < 0.01, *** *p* < 0.001, **** *p* < 0.0001, compared with UV vehicle; NS—not significant between datasets.

To examine whether these vitamin-D-related compounds increased DNA repair, we tested unscheduled DNA synthesis (UDS). UV-irradiated keratinocytes or non-irradiated control cells were treated immediately after with vehicle, 1,25(OH)_2_D_3_ or 24(OH)L_3_ in the presence of 5-ethynyl-20-deoxyuridine (EDU) for 1.5 h. UDS-positive nuclei, a measure of DNA repair under the circumstances of the experiment where the keratinocytes were quiescent [86], were detected as punctate nuclei with fluorescent staining (Figure 3A–C). The UDS incidence was assessed as the percentage of UDS-positive cells in 1000 randomly selected cells. While there was a low incidence of UDS positivity in non-irradiated cells treated with vehicle, 1,25(OH)_2_D_3_ or 24(OH)L_3_ (Figure 3A,D), UV-irradiated 1,25(OH)_2_D_3_- (*p* < 0.001) or 24(OH)L_3_-treated cells (*p* < 0.0001) showed a significantly higher UDS incidence than the UV vehicle-treated cells (Figure 3A,D). UDS was also quantified as the average intensity of the positive fluorescence of nuclei (Figure 3C,E), where low, not significant intensity was observed in UV vehicle-treated cells and non-UV cells. The UV-irradiated cells treated with either 1,25(OH)_2_D_3_ (*p* < 0.0001) or 24(OH)L_3_ (*p* < 0.0001) have significantly increased intensity of EDU incorporation compared with UV-irradiated vehicle-treated cells (Figure 3C,E).

### 3.4. 24(OH)L_3_ Increased ATP Levels Similar to 1,25(OH)_2_D_3_ in UV-Irradiated Human Primary Keratinocytes

DNA repair requires energy [38,87]. Exposure to UV irradiation reduced ATP levels in keratinocytes (Figure 3F). Treatment with 24(OH)L_3_ or 1,25(OH)_2_D_3_ immediately after UV increased the ATP levels measured 1.5 h after UV exposure in keratinocytes (*p* < 0.05) compared with the UV vehicle. Significant differences were not observed between the different concentrations of either 24(OH)L_3_ or 1,25(OH)_2_D_3_. Significant changes in ATP levels were not observed in non-irradiated cells treated with either 24(OH)L_3_ or 1,25(OH)_2_D_3_ compared with the non-UV vehicle (Figure 3F).

### 3.5. 24(OH)L_3_ Increased Glycolysis Similar to 1,25(OH)_2_D_3_ in UV-Irradiated Human Primary Keratinocytes

**Figure 4:**
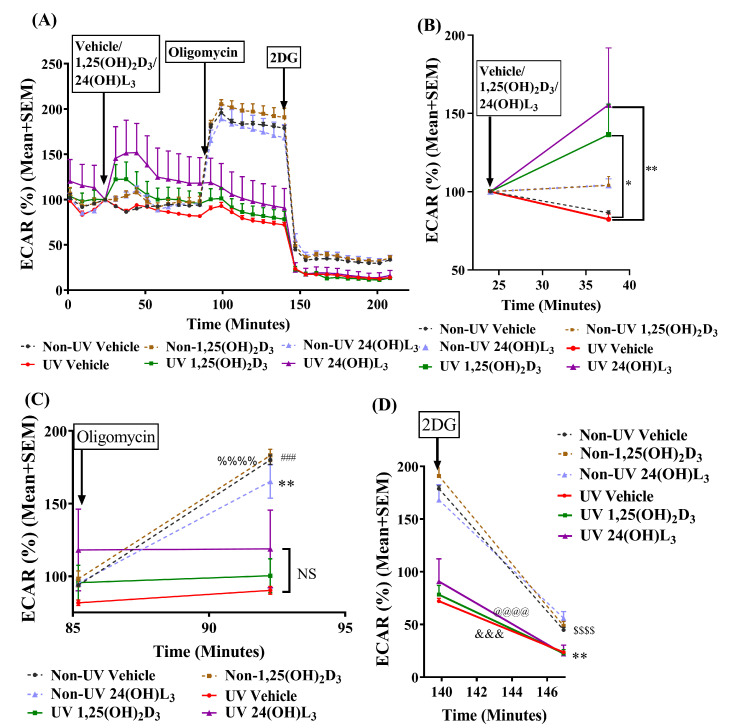
The extracellular acidification rate (ECAR) was increased by treatment with 24(OH)L_3_ or 1,25(OH)_2_D_3_, in UV-irradiated human primary keratinocytes. (**A**) Keratinocytes were subjected to Seahorse XF analysis after UV exposure (UV) or non-UV, and treatments were added at the point of injections, indicated by arrows. Data were normalized using the Seahorse XF Normalization System. (**B**) ECAR following injection of vehicle, 0.1% (*v*/*v*) ethanol, 1 × 10^−8^ M 1,25(OH)_2_D_3_, or 1 × 10^−8^ M 24(OH)L_3_, 25 min after injection (*n* = 14). The ECAR significantly increased in UV 24(OH)L_3_ cells (** *p* < 0.01) and in UV 1,25(OH)_2_D_3_ cells (* *p* < 0.05) compared with the UV vehicle. (**C**) ECAR following 10 µM oligomycin injection. Significantly different from the point of oligomycin injection in the non-UV vehicle (^###^ *p* < 0.001), non-UV-1,25(OH)_2_D_3_ (^%%%%^ *p* < 0.0001), or non-UV-24(OH)L_3_ (** *p* < 0.01), NS, between UV-irradiated cells. (**D**) ECAR after 50 mM 2-deoxy-D-glucose (2-DG) injection. Significantly different from the point of injection, non-UV vehicle, non-UV 1,25(OH)_2_D_3_, non-UV 24(OH)L_3_ (^$$$$^ *p* < 0.0001), UV vehicle (** *p* < 0.01), UV 1,25(OH)_2_D_3_ (^&&&^ *p* < 0.001), and UV 24(OH)L_3_ (^@@@@^ *p* < 0.0001). Results are presented as mean + SEM, compared with the UV vehicle. NS—not significant between datasets.

We previously reported that the oxygen consumption rate (a measure of oxidative phosphorylation) decreased in UV-exposed keratinocytes [79]. Thus, in order to determine the source of the energy in these irradiated and treated keratinocytes, the extracellular acidification rate (ECAR), a measure of glycolysis, was measured using Seahorse XF analysis (Figure 4A). The results indicated that there were no significant changes in the non-irradiated or irradiated keratinocytes over the first 25 min of equilibration after sham or UV exposure prior to the injection of treatments. Significant increases in the ECAR, indicating increased glycolysis, were observed in UV-irradiated, 24(OH)L_3_-treated cells (*p* < 0.01) and in UV-irradiated, 1,25(OH)_2_D_3_-treated cells (*p* < 0.05) (Figure 4B) compared with UV-irradiated vehicle-treated keratinocytes.

The ATP synthase inhibitor, oligomycin, an inhibitor of oxidative phosphorylation, reduced ATP production due to disruption in the electron transport chain [88,89]. The injection of oligomycin significantly increased ECAR in non-UV-exposed vehicle- (*p* < 0.001), 1,25(OH)_2_D_3_- (*p* < 0.0001), and 24(OH)L_3_-treated keratinocytes (*p* < 0.01). This was due to a switch from oxidative phosphorylation to glycolysis and was previously observed in these cells [79]. As seen in Figure 4A,C, the ECAR was unchanged after oligomycin treatment in UV-irradiated cells, consistent with the proposal that oxidative phosphorylation was impaired in these cells after UV. The analogue 2-deoxy-D-glucose (2DG) is a glucose analogue that competitively inhibits glucose uptake and competitively inhibits hexokinase and thus glycolysis [90]. The analogue 2DG significantly reduced the oligomycin-induced glycolysis in non-irradiated cells (*p* < 0.0001), as well as in UV-irradiated vehicle- (*p* < 0.01), 1,25(OH)_2_D_3_- (*p* < 0.001), and 24(OH)L_3_-treated (*p* < 0.001) cells (Figure 4D).

### 3.6. Reductions in UV-Induced CPDs and 8-OHdG by 24(OH)L_3_ or 1,25(OH)_2_D_3_ Were Abolished in the Presence of the Glycolysis Inhibitor (2-Deoxy-D-glucose) but Not by the Oxidative Phosphorylation Inhibitor (Oligomycin) in Human Primary Keratinocytes

**Figure 5:**
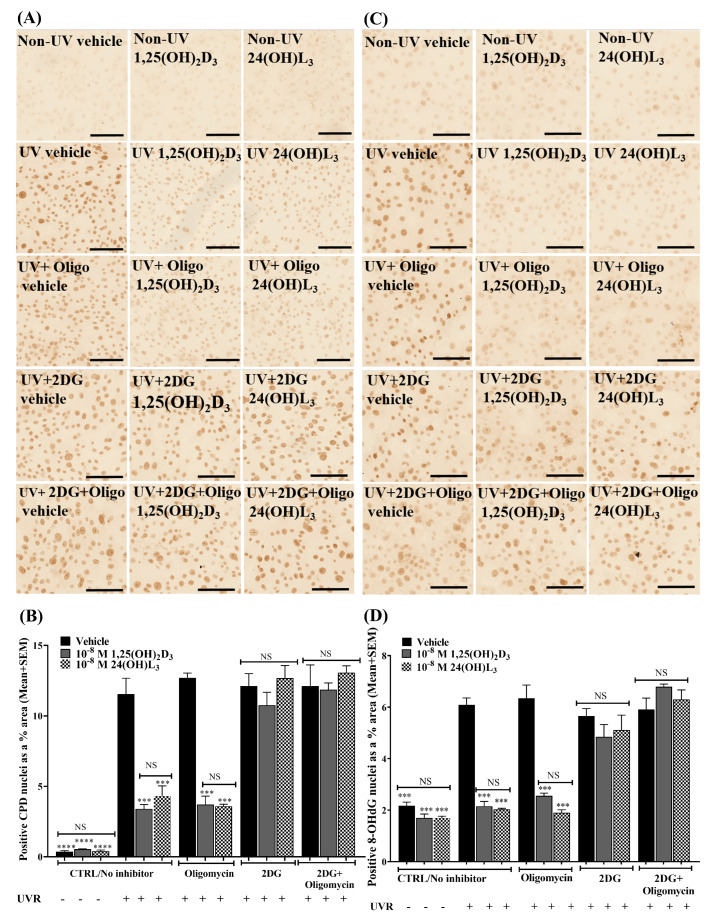
UV-induced CPDs and 8-OHdG reduction by 24(OH)L_3_ or 1,25(OH)_2_D_3_ was abolished by 2-deoxy-D-glucose (2DG) but not by oligomycin in human primary keratinocytes. (**A**) Example bright-field confocal IHC photomicrographs of UV-induced CPDs in keratinocytes treated with vehicle, 0.1% (*v*/*v*) ethanol, 1 × 10^−8^ M 1,25(OH)_2_D_3_, or 1 × 10^−8^ M 24(OH)L_3_ for 1.5 h in the presence or absence of 50 mM 2DG, 10 µM oligomycin, or a combination of 50 mM 2DG and 10 µM oligomycin (scale bar = 100 µm). (**B**) Densitometry presented as CPD-positive nuclei as a % area, 1.5 h after UV irradiation. (**C**) Example bright-field confocal IHC photomicrographs of UV-induced 8-OHdG treated with vehicle, 0.1% (*v*/*v*) ethanol, 1 × 10^−8^ M 1,25(OH)_2_D_3_, or 1 × 10^−8^ M 24(OH)L_3_ for 1.5 h in the presence or absence of 50 mM 2DG, 10 µM oligomycin, or a combination of 50 mM 2DG and 10 µM oligomycin (scale bar = 100 µm). (**D**) Densitometry presented as 8-OHdG-positive nuclei as a % area, 1.5 h after UV irradiation. Mean + SEM; **** *p* < 0.0001 *** *p* < 0.001, when compared with UV vehicle; NS—not significant between datasets.

If increased glycolysis was the source of the energy to increase DNA repair and thus reduce DNA damage in UV-irradiated keratinocytes, then the inclusion of 2-deoxy-D-glucose (2DG) should impair DNA damage reduction, while the addition of oligomycin should have no effect. As previously observed, the addition of 24(OH)L_3_ or 1,25(OH)_2_D_3_ significantly reduced the CPDs (Figure 5A,B) and 8-OHdG (Figure 5C,D) in human primary keratinocytes after UV (*p* < 0.001). Treatment with 2DG abolished the reduction of UV-induced CPDs and 8-OHdG by 24(OH)L_3_ or 1,25(OH)_2_D_3_ measured 1.5 h after irradiation. Treatment with oligomycin did not affect the reduction of UV-induced CPDs or 8-OHdG with 24(OH)L_3_ or 1,25(OH)_2_D_3_ after 1.5 h. Treatment with the combination of 2DG and oligomycin also abolished the reduction of UV-induced photolesions in the presence of 24(OH)L_3_ and 1,25(OH)_2_D_3_ to a similar extent as 2DG alone (Figure 5A–D).

### 3.7. 24(OH)L_3_ Reduced Reactive Oxygen Species (ROS) Similar to 1,25(OH)_2_D_3_ in UV-Irradiated Human Primary Keratinocytes

**Figure 6:**
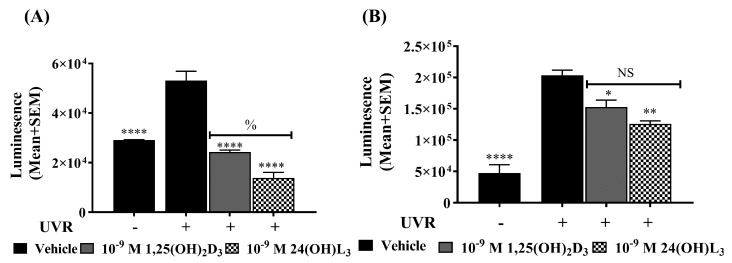
Reactive oxygen species (ROS) were reduced in UV-irradiated human keratinocytes treated with either 24(OH)L_3_ or 1,25(OH)_2_D_3_. (**A**) Human primary keratinocytes were irradiated and immediately treated with either vehicle, 0.1% (*v*/*v*) ethanol, 1 × 10^−9^ M 1,25(OH)_2_D_3_, or 1 × 10^−9^ 24(OH)L_3_, and ROS levels were measured using the ROS-Glo™ commercial assay after (**A**) 15 min or (**B**) 90 min after irradiation. Results were from a single experiment performed in triplicate, representative of two separate experiments with similar results. Mean + SEM; **** *p* < 0.0001, ** *p* < 0.01, * *p* < 0.05 when compared with UV vehicle, ^%^ *p* < 0.05 for UV-1,25(OH)_2_D_3_ vs UV-24(OH)L_3_ after 15 min; NS—not significant between datasets.

The generation of ROS by UV exposure directly causes oxidative DNA damage [91] and inhibits DNA repair [34]. As expected, UV irradiation increased the ROS levels in keratinocytes (Figure 6A,B). Treatment with either 24(OH)L_3_ or 1,25(OH)_2_D_3_ immediately after irradiation significantly reduced ROS levels in keratinocytes (*p* < 0.0001) compared with the UV vehicle, when measured either 15 min after UV (Figure 6A) or 1.5 h after UV for 24(OH)L_3_ (*p* < 0.01) and for 1,25(OH)_2_D_3_ (*p* < 0.05) (Figure 6B).

### 3.8. Topical Treatment with 1,25(OH)_2_D_3_ or 24(OH)L_3_ Increased XPC and XPA in UV-Irradiated Skin Explants

**Figure 7:**
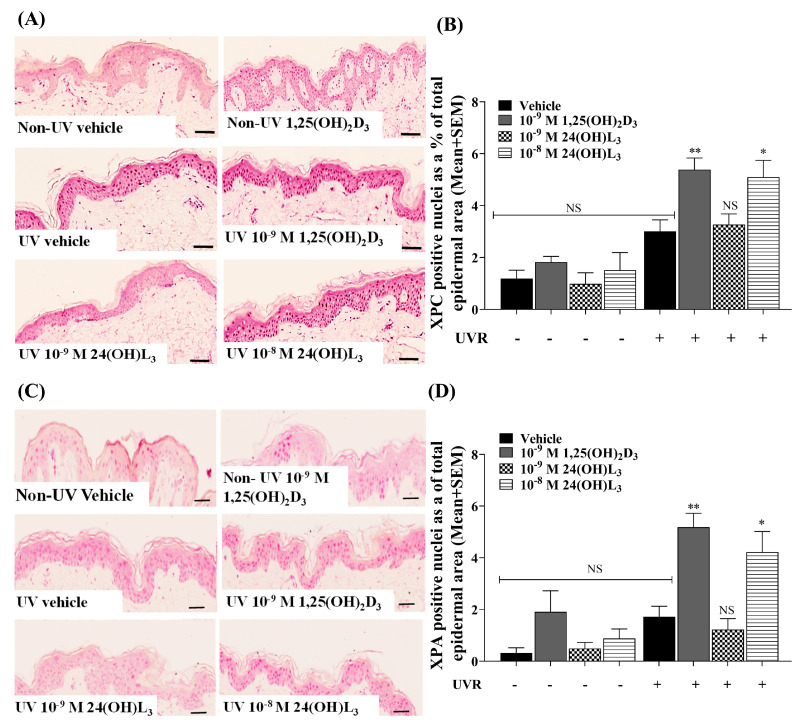
Topical treatment of human skin explants with 1,25(OH)_2_D_3_ or 24(OH)L_3_ increased NER proteins XPC and XPA after UV. (**A**) Example bright-field confocal IHC photomicrographs of UV-induced XPC expression or (**C**) XPA expression in human skin explants, topically treated with vehicle, 0.1% (*v*/*v*) ethanol, 1 × 10^−9^ M 1,25(OH)_2_D_3_, 1 × 10^−8^ M, or 1 × 10^−9^ M 24(OH)L_3_ for 3 h (scale bar = 50 µm). Densitometry presented as (**B**) XPC-positive nuclei as a % total epidermal area and (**D**) XPA-positive nuclei as a % total epidermal area. Results are from a single experiment performed using five skin explants per treatment, representative of three separate experiments with similar results. Mean + SEM; ** *p* < 0.01, * *p* < 0.05, when compared with UV vehicle; NS—not significant between datasets−.

If CPD repair by the nucleotide excision repair (NER) pathway were increased, it would be expected that an increase in key NER proteins such as XPC and XPA would be observed. In UV-irradiated, vehicle-treated skin explants, there was a small but not significant increase in XPC (Figure 7A,B) or XPA (Figure 7C,D), compared with non-irradiated skin explants. In contrast, significant increases in XPC (*p* < 0.01) (Figure 7B) and XPA (*p* < 0.01) (Figure 7D) were observed in skin explants treated topically with 1 × 10^−9^ M 1,25(OH)_2_D_3_. Treatment with 1 × 10^−8^ M 24(OH)L_3_ significantly increased both XPC and XPA (*p* < 0.05), but there was no significant increase in either protein after treatment with 1 × 10^−9^ M 24(OH)L_3_ (Figure 7B,D).

### 3.9. Reductions of UV-Induced CPDs by 1,25(OH)_2_D_3_ and 24(OH)L_3_ Were Abolished by Knockdown of XPC or XPA

**Figure 8:**
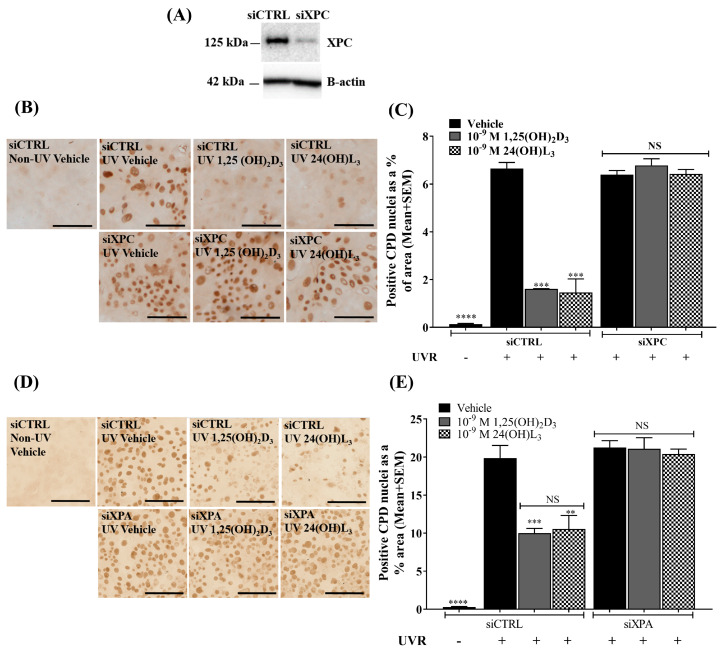
UV-induced CPD reduction by 1,25(OH)_2_D_3_ or 24(OH)L_3_ was abolished by XPC or XPA knockdown in human primary keratinocytes. (**A**) Western blot showing the level of XPC protein in human primary keratinocytes following transfection with siCTRL or siXPC for 48 h. (**B**) Representative photomicrographs of IHC staining of UV-induced CPDs treated after UV with vehicle, 0.1% (*v*/*v*) ethanol, 1 × 10^−9^ M 1,25(OH)_2_D_3_, or 1 × 10^−9^ M 24(OH)L_3_ after transfection with siCTRL or siXPC. Dark brown staining indicates the presence of CPD-positive nuclei (scale bar = 100 μm). (**C**) Image analysis of IHC images in panel B where values were calculated as CPD-positive nuclei as a % area. (**D**) Representative photomicrographs of immunohistochemical staining of UV-induced CPDs treated after UV with vehicle, 0.1% (*v*/*v*) ethanol, 1,25(OH)_2_D_3_, or 24(OH)L_3_ after transfection with siCTRL or siXPA. Dark brown staining indicates the presence of CPD-positive nuclei (scale bar = 100 μm). (**E**) Image analysis of IHC images in panel D presented as CPD-positive nuclei as a % area. Results are from a single experiment performed in triplicate, representative of three separate experiments with similar results. Mean + SEM; **** *p* < 0.0001, *** *p* < 0.001, ** *p* < 0.01, when compared with UV siCTRL vehicle. NS—not significant between datasets.

In order to see whether the NER proteins indeed were critical for the increased repair of CPDs, the keratinocytes were first transfected with small interfering RNA targeted to XPC mRNA (siXPC) or with a non-directed siRNA sequence (siCTRL), as described in [92]. XPC knockdown by siRNA was verified by Western blot (Figure 8A). Human primary keratinocytes were irradiated 48 h after siRNA transfection and immediately treated with either vehicle or 1,25(OH)_2_D_3_ or 24(OH)L_3_ each at 1 × 10^−9^ M for 3 h. Photomicrographs of immunohistochemical staining for UV-induced CPDs are presented in Figure 8B. The UV-irradiated vehicle-treated cells showed a significant increase in CPDs compared with non-UV cells in cells transfected with a non-directed sequence (siCTRL) (*p* < 0.0001). Significant reductions in UV-induced CPDs were observed in cells treated with 1,25(OH)_2_D_3_ and 24(OH)L_3_ (*p* < 0.001 for both) in the siCTRL samples. Reductions of CPDs with 1,25(OH)_2_D_3_ or 24(OH)L_3_ were abolished in the presence of cells transfected with siRNA directed at XPC (siXPC) (Figure 8B,C).

A similar study was carried out in keratinocytes transfected with siRNA targeted to XPA mRNA (siXPA) or a non-directed siRNA sequence (siCTRL). Again, UV-irradiated vehicle-treated human primary keratinocytes showed significant increases in CPDs compared with non-irradiated cells in siCTRL samples (Figure 8D,E; *p* < 0.0001). UV-irradiated keratinocytes treated with 1,25(OH)_2_D_3_ or 24(OH)L_3_ for 3 h had significantly reduced UV-induced CPDs (*p* < 0.001 or *p* < 0.01 respectively) in siCTRL samples. The reduction of CPDs by 1,25(OH)_2_D_3_ or 24(OH)L_3_ was abolished in cells transfected with siRNA directed at XPA (Figure 8D,E).

## 4. Discussion

The treatment with 1,25(OH)_2_D_3_ has been shown to reduce UV-induced DNA damage in skin in many studies [52,55,57,76,79,85,93,94,95]. The current study investigated the photoprotective properties of one of the most abundant “over-irradiation” metabolites, 24(OH)L_3_, in comparison with 1,25(OH)_2_D_3_. This study tested only 24(OH)L_3._ While further metabolism of 24(OH)L_3_ by other CYP enzymes known to metabolize vitamin-D-related compounds is theoretically possible, there is no precedent for CYP24A1, which metabolizes 25-hydroxyvitamin D and 1,25(OH)_2_D_3_ to their 24-hydroxylated derivatives, to act on a sterol with an intact B-ring. Even though it is possible that CYP27A1 acts on 24(OH)L3, as it can act on other lumisterol compounds (R. Tuckey, personal communication), it is doubtful that there is enough CYP27A1 in skin to make a difference. Whether any metabolism of 24(OH)L3 by CYP27A1 changes biological activity is also unknown. UV-induced CPDs, as well as 8-OHdG, were reduced in a concentration-dependent manner by the addition of 24(OH)L_3_ immediately after UV exposure, similar to 1,25(OH)_2_D_3_. In keratinocytes, both compounds were effective at a low concentration of 1 × 10^−10^ M, but in human skin explants, the minimal effective dose of 1,25(OH)_2_D_3_ to reduce both CPDs and 8-OHdG was 10-fold higher at 1 × 10^−9^ M. The minimal effective dose of 24(OH)L_3_ in skin explants was a further 10-fold higher. It is likely that the thickness of epidermis in the human skin reduced the penetration of both compounds, with that of 24(OH)L_3_ reduced to a greater extent than that of 1,25(OH)_2_D_3_, although this is yet to be investigated in more detail.

Our results indicated that at least part of the reason for the reduction in DNA damage with 24(OH)L_3_ was an increase in DNA repair as evidenced by the increased intensity and number of UDS-positive nuclei in the presence of 24(OH)L_3_ compared with the UV vehicle in UV-exposed keratinocytes. These increases were similar to those seen with 1,25(OH)_2_D_3_. As illustrated in Figure 3A,B, DNA repair was observed mainly in the nuclei of keratinocytes, although the repair of mitochondrial DNA cannot be entirely excluded. We have previously reported increased DNA repair after UV with 1,25(OH)_2_D_3_ [79]. DNA repair is an energy-intensive metabolic process. UV radiation damages mitochondria, and the damage impairs their main function, cellular respiration [31,96,97]. An earlier study by our group indicated that oxygen consumption rates, which are measures of oxidative phosphorylation, decreases after UV exposure [79]. As shown here, oligomycin, an inhibitor of oxidative phosphorylation [88,89], had no effect on the ECAR response of the UV exposed cells due to inactive oxidative phosphorylation. As expected, the treatment of the keratinocyte cultures with oligomycin also had no effect on the reduction of UV-induced DNA damage with 24(OH)L_3_ or the hormonal form of vitamin D.

Although ATP levels were reduced after UV, treatment with either 1,25(OH)_2_D_3_ or 24(OH)L_3_ increased ATP. We looked at an alternative source of energy, glycolysis. There were no significant changes in glycolysis, as measured by ECAR in UV-irradiated cells injected with vehicle. In contrast, glycolysis was increased by both 1,25(OH)_2_D_3_ and 24(OH)L_3_ in UV-irradiated keratinocytes, as reported previously for 1,25(OH)_2_D_3_ [79]. Oligomycin increased ECAR in non-irradiated cells, indicating that oxidative phosphorylation was still intact in these cells, unlike the UV-exposed cells, as energy production in the cell switched from oxidative phosphorylation to glycolysis upon the addition of the oxidative phosphorylation inhibitor in the non-irradiated keratinocytes. As seen in Figure 4A, the addition of 2-deoxy-D-glucose suppressed glycolysis/ECAR in all keratinocytes. The addition of 2-deoxy-D-glucose to the keratinocytes also abolished the reduction in UV-induced CPDs and 8-OHdG by 1,25(OH)_2_D_3_ or 24(OH)L_3_, consistent with the proposal that the active glycolysis produced by treatment with 1,25(OH)_2_D_3_ or 24(OH)L_3_ was critical for the reduction in UV-induced DNA damage.

The electron transport chain in mitochondria is the main source of intrinsic ROS production in cells [98]. UV irradiation increases ROS production, and the damage to mitochondria by UV may further increase cellular ROS levels [99,100]. Increased ROS levels were observed in UV-irradiated keratinocytes at 15 min and 1.5 h post-UV. The UV-irradiated keratinocytes treated with 1,25(OH)_2_D_3_ or 24(OH)L_3_ had significantly reduced ROS, indicating that these compounds could contribute to a reduction of ROS levels after UV. While one explanation could be just that 1,25(OH)_2_D_3_ or 24(OH)L_3_ slowed oxidative phosphorylation, we have previously reported that the treatment of keratinocytes with 1,25(OH)_2_D_3_ increased an early form of mitophagy [101], leading to mitochondrial repair [79] and reduced ROS. There are data indicating that 1,25(OH)_2_D_3_ via the vitamin D receptor (VDR) is able to bind mitochondrial DNA and affect the transcription of oxidative phosphorylation subunits [102], while silencing of the VDR in various cell lines caused excessive ROS generation [103]. There is also evidence that 1,25(OH)_2_D_3_ activates the Nrf2–Keap1 antioxidant pathway, which ameliorates oxidant stress [104,105,106], and that 1,25(OH)_2_D_3_, lumisterol, and even tachysterol hydroxyderivatives do so in UV-exposed keratinocytes [107,108] and thus potentially reduce oxidative DNA damage. It is highly likely that reduced ROS levels as a result of treatment with 1,25(OH)_2_D_3_ or 24(OH)L_3_ directly contribute to reduced 8-OHdG levels [91] and nitrosative stress [109] in UV-irradiated keratinocytes. Importantly, with reduced ROS and reactive nitrogen species [53,76], there will be less damage to cellular proteins, including proteins involved in DNA repair, and thus more effective repair [34,110]

Since the abovementioned mechanisms may collectively contribute to the increased DNA repair after UV, we explored two proteins involved in the nucleotide excision repair pathway for CPDs. XPC is an initial damage recognition protein in the GG–NER pathway, while XPA helps to verify the DNA damage and stabilize the DNA as it is repaired and is required for both GG–NER and TC–NER [111]. XPC and XPA accumulate at the sites of DNA damage [112]. UV-induced CPDs cause only a small distortion in the DNA helix and so are dependent for detection on the binding of XPE, a complex of damaged DNA binding proteins 1 and 2 (DDB1 and DDB2) [38]. DDB recruits XPC, which together with XPA is required for the successful initiation of NER [113]. In the current study, the XPC and XPA protein levels were increased in response to treatment with the lumisterol-derived 24(OH)L_3_ as well as 1,25(OH)_2_D_3_ in UV-irradiated human explants. Furthermore, knockdown of either XPC or XPA abolished the reduction in CPDs normally seen after the addition of either 1,25(OH)_2_D_3_ or 24(OH)L_3_, indicating key roles for these proteins in this process. No other changes in DNA repair proteins after 1,25(OH)_2_D_3_ or 24(OH)L_3_ treatment were examined in this study. It has previously been reported that the treatment of human keratinocytes in culture with 1,25(OH)_2_D_3_ upregulated mRNA for XPC and DDB2 [114]. In an earlier study, both XPC and DDB2 protein levels were significantly increased in human skin biopsies treated topically with 1,25(OH)_2_D_3_ at non-UV or UV-irradiated sites [115].

A possible mechanism for the increase in XPC involves phosphatase and tensin homolog deleted on chromosome ten (PTEN). PTEN is tumor suppressor and acts as a negative regulator of the oncogenic acutely transforming retrovirus AKT8 in the rodent T-cell lymphoma (AKT) pathway [116]. It has been reported that UVB down-regulates PTEN in primary human keratinocytes and in mouse skin in an AKT- and extracellular-signal-regulated kinase (ERK)_1/2_-dependent manner [117]. This downregulation of PTEN reduces global genomic NER, through reductions in XPC [118]. More recent studies by Shariev et al. showed that exposure to solar-simulated UV decreased PTEN levels in Skh:hr1 hairless mouse skin [119], as well as primary melanocytes and melanoma cells [120]. The addition of 1,25(OH)_2_D_3_ inhibited this UV-induced depletion of PTEN [119], which likely explains the suppression of AKT phosphorylation after UV by 1,25(OH)_2_D_3_ [79].

In all this, although vitamin D_3_ and lumisterol hydroxyderivatives have been shown to bind to retinoid receptors [71] and to liver X receptors [121], the vitamin D receptor (VDR) in skin and the endoplasmic reticulum protein p57 (ERp57) are clearly key for the reductions in DNA damage by 1,25(OH)_2_D_3_ or 24(OH)L_3_. The reductions in both CPDs and 8-OHdG after UV by either 1,25(OH)_2_D_3_ or 24(OH)L_3_, as well as a CYP11A1 derivative of vitamin D_3_, 20(OH)D_3_, were abolished with the knockdown of either ERp57 or VDR [85,122]. VDR-null mice show increased UV-induced DNA damage [123], as well as increased skin tumor formation after UV or chemical carcinogenesis [123,124]. VDR mRNA expression and protein levels were reduced in UVB-irradiated keratinocytes [125]. When human keratinocytes were treated with 24(OH)L_3_, VDR mRNA and protein levels increased in both UV-irradiated and non-irradiated cells [71].

## 5. Conclusions

These data collectively indicated that the major over-irradiation metabolite, 24(OH)L_3_, derived from lumisterol through the actions of CYP11A1 in skin, contributed to the reduction of UV-induced damage in a manner similar to the vitamin D hormone, 1,25(OH)_2_D_3_, in keratinocytes and the whole skin. Other CYP11A1 derivatives produced in skin from both vitamin D_3_ and lumisterol [68,126,127] also reduced UV-induced DNA damage [71,122,126,128]. These findings are consistent with the proposal that this range of UV-generated, 7-dehydrocholesterol-derived, CYP11A1-produced compounds also help reduce UV-induced DNA damage, which is a key precursor to skin tumor development. This proposal would provide an explanation for the reported observations that mice with a knockout of CYP27B1, which is solely responsible for the production of 1,25(OH)_2_D_3_ and so cannot produce 1,25(OH)_2_D_3_, nevertheless are not more susceptible to UV-induced skin tumors than wild-type mice [129], whereas VDR knockout mice show much greater susceptibility to UV-induced photocarcinogenesis [123].

## Data Availability

The data that support the findings of this study are available from the corresponding author upon reasonable request. Some data may not be made available because of privacy or ethical restrictions.
